# Topological diversity of chromatin fibers: Interplay between nucleosome repeat length, DNA linking number and the level of transcription

**DOI:** 10.3934/biophy.2015.4.613

**Published:** 2015-11-03

**Authors:** Davood Norouzi, Ataur Katebi, Feng Cui, Victor B. Zhurkin

**Affiliations:** 1 Laboratory of Cell Biology, National Cancer Institute, NIH Bethesda, MD 20892, USA; 2 Thomas H. Gosnell School of Life Sciences, Rochester Institute of Technology, 85 Lomb Memorial Drive, Rochester, NY 14623, USA

**Keywords:** chromatin fiber, nucleosome repeat length, DNA linking number, DNA topology, regulation of transcription

## Abstract

The spatial organization of nucleosomes in 30-nm fibers remains unknown in detail. To tackle this problem, we analyzed all stereochemically possible configurations of two-start chromatin fibers with DNA linkers L = 10–70 bp (nucleosome repeat length NRL = 157–217 bp). In our model, the energy of a fiber is a sum of the elastic energy of the linker DNA, steric repulsion, electrostatics, and the H4 tail-acidic patch interaction between two stacked nucleosomes. We found two families of energetically feasible conformations of the fibers—one observed earlier, and the other novel. The fibers from the two families are characterized by different DNA linking numbers—that is, they are topologically different. Remarkably, the optimal geometry of a fiber and its topology depend on the linker length: the fibers with linkers L = 10*n* and 10*n* + 5 bp have DNA linking numbers per nucleosome Δ*Lk* ≈ −1.5 and −1.0, respectively. In other words, the level of DNA supercoiling is directly related to the length of the inter-nucleosome linker in the chromatin fiber (and therefore, to NRL). We hypothesize that this topological polymorphism of chromatin fibers may play a role in the process of transcription, which is known to generate different levels of DNA supercoiling upstream and downstream from RNA polymerase. A genome-wide analysis of the NRL distribution in active and silent yeast genes yielded results consistent with this assumption.

## 1. Introduction

Eukaryotic DNA is spatially organized in a hierarchical manner, the first level being the chain of nucleosomes connected by DNA linkers (“beads on a string”). Stereochemical details of the second level of DNA organization, the so-called 30-nm fiber, still remain a subject of debate [1–4], despite the fact that the structure of the nucleosome was determined at a high resolution more than 10 years ago [5].

During the past decade there has been a significant progress in the structural studies of chromatin, starting with the seminal studies of Richmond and co-authors, who first observed the two-start fibers (Dorigo et al. [6] and Schalch et al. [7]). Their results, together with the high-resolution Cryo-EM data obtained by Song et al. [8] strongly support the two-start organization of chromatin fibers for relatively short linkers L = 20, 30 and 40 bp. However, the electron microscopy (EM) images presented by Rhodes and co-authors [9,10] suggest that for L = 50 bp and longer, the one-start solenoid (or interdigitaded structure) is more stable, especially in the presence of linker histones.

All these structures were obtained for arrays of strongly positioned “601” nucleosomes [11], with the nucleosome repeat length (NRL) varying from 167 to 237 bp in increments of 10 bp. Assuming that the nucleosome core is 147 bp [5], this means that the linker length varies from 20 to 90 bp—that is, L belongs to the {10*n*} series. On the other hand, it is known that *in vivo*, the linker sizes are close to {10*n* + 5} values, at least for yeast and mouse [12,13]; in particular, L = 15 bp is predominant in the baker’s yeast chromatin [12,14,15]. Thus, the structural data mentioned above correspond to linker lengths with occurrences *in vivo* that are relatively small.

Only recently, Grigoryev and co-authors analyzed nucleosomal arrays with linker lengths belonging both to the series {10*n*} and {10*n* + 5} (Correll et al. [16]). Using sedimentation and EM visualization, they demonstrated that fibers with L = 25 bp (NRL = 172 bp) have less propensity to fold in a compact state compared to L = 20 or 30 bp. The increased fiber “plasticity” observed for L = 10*n* + 5 may be functionally significant because the {10*n* + 5} values are frequently found *in vivo*.

The distinct folding pathways observed for fibers with L = 10*n* and L = 10*n* + 5 [16] indicate that these fibers may have different configurations, and in particular, different spatial arrangements of DNA linkers. This polymorphism may be related to the early studies by Worcel et al. [17], Woodcock et al. [18] and Williams et al. [19] whose space-filling models of the chromatin fiber were presented with the DNA linking number Δ*Lk* varying from −1 to −2 per nucleosome, depending on the DNA trajectory. (At that time, the Δ*Lk* values were not calculated precisely, but rather were estimated qualitatively.)

Generally speaking, this variability of Δ*Lk* can be attributed to the existence of different fiber topoisomers corresponding to different levels of DNA supecoiling, which may have far-reaching biological implications, from local protein-DNA interactions [20] to large-scale regulation of transcription [21]. However, the topological properties have not been analyzed in computational studies of regular chromatin fibers published recently [22,23]. Therefore, in this study, we performed a thorough computational analysis of regular two-start fibers, focusing on their topology.

Earlier, we presented results of computations for relatively short linkers L < 40 bp [24]. Here, we analyze chromatin fibers with the linker lengths varying from 10 to 70 bp, and confirm the main conclusions made earlier. First, we describe two families of conformations—one (denoted T2) represents the structures visualized in the X-ray and Cryo-EM studies [7,8], and the other (denoted T1) comprises “novel” structures not observed experimentally so far. The fibers from the two families are characterized by different Δ*Lk* values—that is, they are topologically different. Second, there is a strict relationship between the type of topoisomer and the linker length: for linkers L = 10*n*, the “old” topoisomer T2 is energetically optimal, whereas for linkers L = 10*n* + 5 the “novel” topoisomer T1 is more favorable. These features are valid for the whole interval of the linker lengths. There are certain differences between the “short” and “long” linkers, however. In particular, for linkers L > 50 bp the energy minima become more shallow and the energy barriers between the T1 and T2 minima less pronounced. This tendency is consistent with the experimental data mentioned above [9,10].

Next, we analyze how the DNA linking number Δ*Lk* depends on the finite size of chromatin fiber. The obtained results are critical for quantitative interpretation of the experimental results on the level of DNA supercoiling in circular closed DNA (ccDNA) with reconstructed nucleosomal arrays [25,26,27].

Finally, we present convincing evidence that the NRL observed *in vivo* correlates with the level of gene expression in yeast. Since the NRL (and the inter-nucleosome linker L) define the DNA supercoiling density, we consider this particular result as a reflection of the more general interrelationship between the “local” topological polymorphism of chromatin fibers and the “global” topological changes in DNA occurring during transcription.

## 2. Methods

### 2.1. Geometry of chromatin fibers and energy calculation

**Figure 1:**
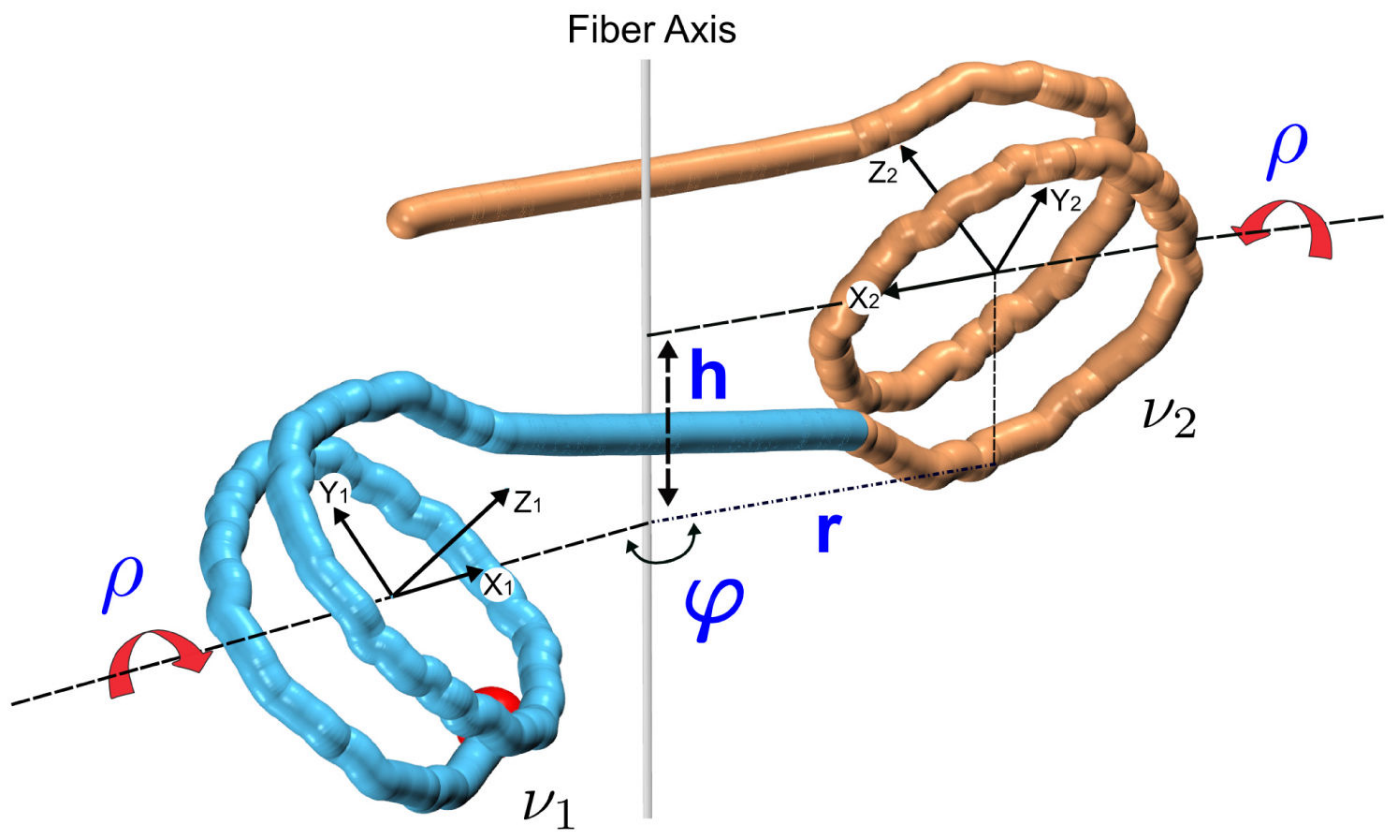
Definition of the four parameters describing the conformation of a regular symmetrical chromatin fiber [24] These are three cylindrical parameters h (rise), r (radius) and polar angle *φ*, and the internal inclination angle ρ. Each nucleosome is associated with a right-handed coordinate frame in which axis Z represents the superhelical axis of the nucleosome (calculated as described earlier [62]), axis X points toward the nucleosome dyad, and axis Y is perpendicular to X and Z. The inclination angle *ρ* defines rotation of a nucleosome around the dyad axis X. The entry point of nucleosome ν1 is shown as a red ball. The image was prepared with the VMD software package [63].

Positions of nucleosomes in a regular symmetrical fiber can be described by four parameters (Figure 1). (The chromatin fiber is called regular if all inter-nucleosome linkers have the same conformation and symmetrical if its structure does not change when it is turned upside down.) The three cylindrical coordinates define positions of the nucleosome centers: radius *r*, rise *h* and polar angle *φ*. This angle determines the handedness of the fiber and number of stacks. For instance, the left-handed two-stack fibers correspond to *φ* between 150° and 180°. The fourth parameter, *ρ*, defines inclination of nucleosomes relative to the fiber axis. This corresponds to a rotation around the dyad axis X (Figure 1). This parameter plays a special role in our analysis because it determines the twist in linker DNA and the topological properties of the fiber in general [24].

The nucleosome core particles (DNA and histones) are considered to be rigid in our computations, with the coordinates taken from the high-resolution crystal structure [5].

To find the optimal conformation of the DNA linker connecting two nucleosomes (Figure 1) we use a “mesoscopic” approach [28] in which DNA is modeled at the level of dimeric steps, and its trajectory is described by the six base-pair step parameters Twist, Roll, Slide, etc. [29]. The elastic energy of the linker DNA deformation is calculated using the knowledge-based potential functions introduced by Olson et al. [30]. The stiffness constants, including the cross correlations (such as Twist-Roll) are taken as averages for all 16 dinucleotides. For rest-state values, we use the average helical parameters of B-DNA: Twist = 34.5° and Rise = 3.35 Å; the other rest-state values, such as Slide, are taken to be zero. The DNA linker minimization is nested in the outer cycle in which the total energy of the nucleosome fiber is minimized as a function of the four parameters defining the fiber configuration (Figure 1).

Four energy terms are considered in our calculations: the elastic energy of linker DNA (see above), the repulsive and electrostatic interactions (calculated using the Coulomb potential with 30 Å distance cutoff), and the H3 tail—acidic patch interactions between the adjacent nucleosomes, which are modeled phenomenologically as described earlier [24].

### 2.2. DNA topology

**Figure 2:**
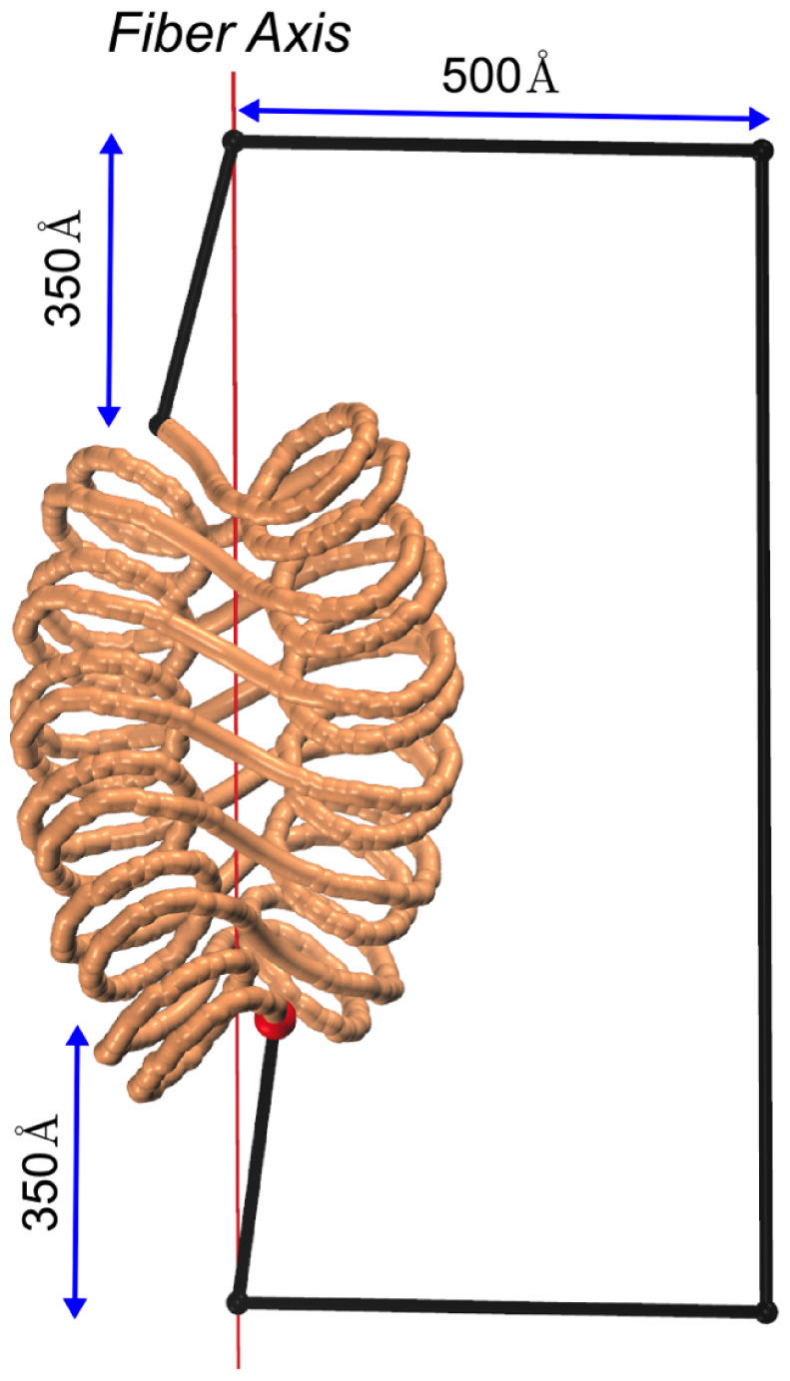
Closing the fiber ends to compute the DNA writhing, *Wr* Two points are chosen on the fiber axis, 350 Å above and below the fiber periphery. The other two points are obtained by shifting the first two points by 500 Å in the plane defined by the fiber axis and the entry point shown as a red ball. The direction of shift is perpendicular to the fiber axis.

The DNA topology is described by three parameters: Δ*Tw* (the change in DNA twisting), DNA writhing, *Wr,* and the change in the linking number, Δ*Lk* (compared to the relaxed state of DNA) [31,32,33]. They are related by the well-known equation: Δ*Lk* = *Wr* + Δ*Tw*. This equation is valid for closed circular DNA; therefore, we need to find an effective way to build the closed DNA trajectory. We add four extra points connecting the ends of DNA in a way that these points remain in one plane with the fiber axis and the closing chain does not pass through nucleosomes (Figure 2). In this case, the DNA fragments connecting these four points do not introduce additional writhing because they are in the same plane. Note that our approach is similar to that used by Fuller to close the ends of a ribbon wound in a regular superhelix (see Figure 4 in [31]).

To calculate the DNA writhing, we use the quadrangle method that was first derived by Levitt [34] for the description of protein folding and was later used for DNA by Klenin and Langowski [35]. The DNA trajectory is represented by a polygonal chain with the vertex points at the centers of base pairs. The DNA twisting is determined using the Euler angle formalism [36,37] implemented in CompDNA [38], 3DNA [39] and SCHNAaP [40] software.

## 3. Results

### 3.1. Energetically optimal conformations

As mentioned above, the two series of linker length, L = 10*n* and 10*n* + 5, have been the focus of investigators, the former because chromatin fibers with L = 10n are more stable *in vitro*, and the latter because linkers L = 10*n* + 5 are observed *in vivo*. Therefore, we paid most attention to these values of L.

**Figure 3:**
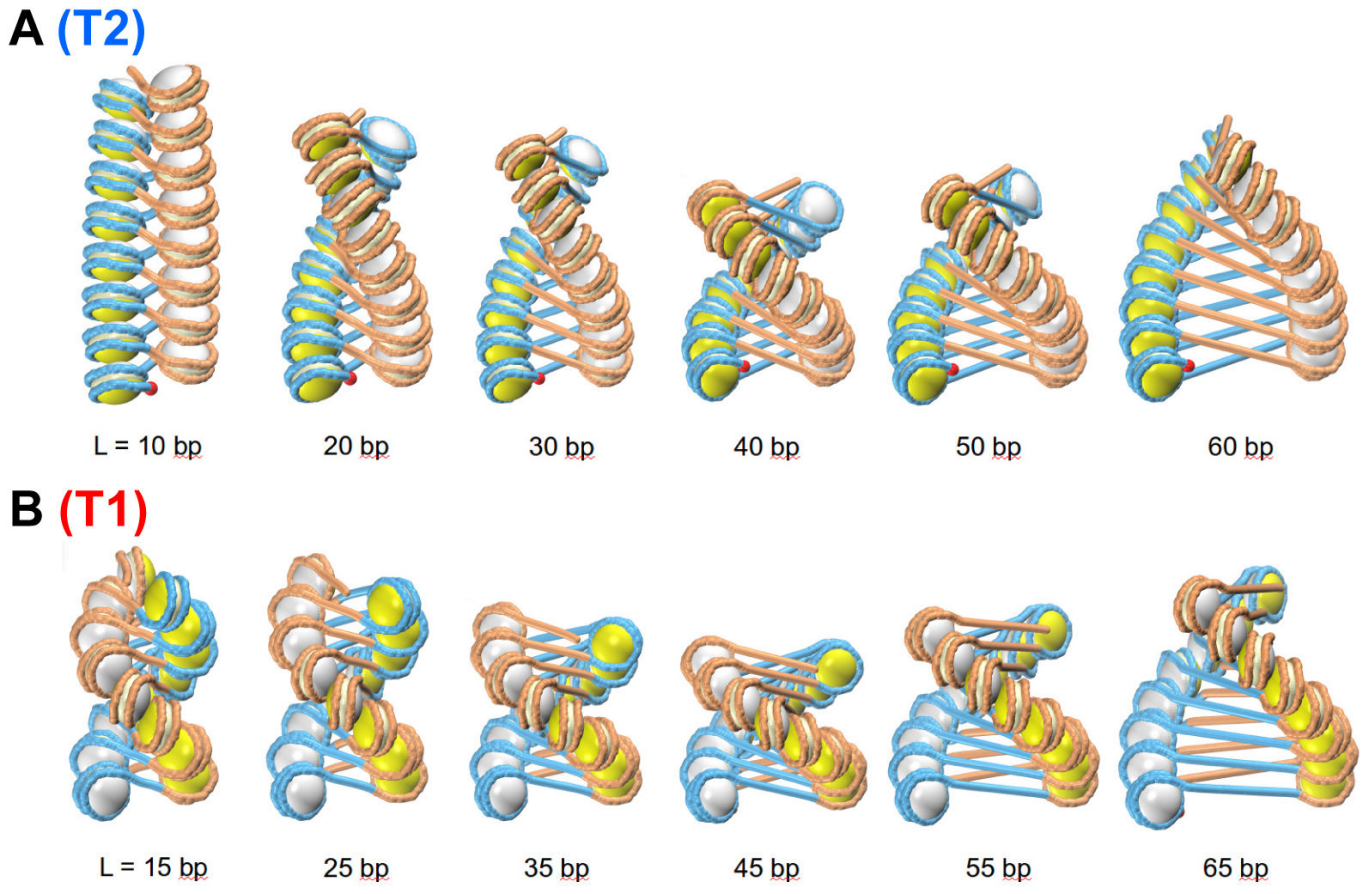
Two-start chromatin fibers for variable linker sizes Optimal structures are shown for the two series of linker lengths, *i.e.* for L = 10*n* (A) and L = 10*n* + 5 (B). For the {10*n*} series, the inclination angle *ρ* varies from −80° to −30°. For the {10*n* + 5} series, the angle *ρ* changes from 80° to 110°. In this case, the nucleosomes are “almost” parallel to the fiber axis. Note that the fiber dimensions vary significantly for both series, but overall the fibers for L = 10*n* are more extended than for L = 10*n* + 5. The most compact forms (with the smallest rise) occur for L = 40–45 bp. For the shorter linkers L = 10–25 bp, in order to avoid clashes between linkers and nucleosomes, the rise is higher. In both series the diameter increases linearly with the linker length. The structures for L = 20, 25 and 30 bp were presented earlier [24]. The fibers shown in (A) and in (B) belong to different topological families, denoted T2 and T1, respectively. As shown in Figure 5, the two series of structures are distinguished by the DNA linking number.

Our computations demonstrate significant variability of two-start chromatin fibers (Figure 3), which is the result of interplay between the linker DNA twisting (naturally depending on the linker length) and the inclination of nucleosomal disks (Figure 1). For example, note the nearly two-fold increase in diameter of fibers accompanying a linker increase from 10–15 to 60–65 bp. This trend is to be expected because the linker length dictates the diameter. On the other hand, the fiber extension clearly visible for the short linkers, L = 10–20 bp, is apparently favorable, as it helps to avoid steric clashes between nucleosomes when their orientation is “almost horizontal”.

Comparison of our calculated structures with those observed experimentally is possible only for L = 20 to 40 bp (Supplementary Figure S1). Our optimal structures are remarkably similar to those visualized by Cryo-EM [8] for L = 30 and 40 bp in terms of the super-helical parameters shown in Figure 1. For example, the parameter rise is 24 Å and 22.5 Å in the experimental structures, while our computations predict the values 25 Å and 20 Å (for L = 30 and 40 bp, respectively). In the case of L = 20 bp, we cannot directly compare our regular fiber with the irregular dinucleosome crystal structure solved by Schalch et al. [7]. The “direct” model built in that study had a low rise of 17 Å and several “steric overlaps” [7]. Therefore, we used EM images presented by Routh et al. [9] and Correll et al. [16] and estimated the rise to be ~ 25 Å, which is close to our predicted rise = 27 Å.

For the L = 10*n* + 5 series, we predicted a family of novel structures that are clearly different from the fibers with L = 10*n* (Figure 3B). Note the “nearly vertical” orientation of nucleosomes in the novel structures—they are rotated by more than 90° compared to the known structures for L = 20–30 bp, so that the red balls indicating “entry points” are invisible in this projection. Since there are no experimentally observed high-resolution fiber structures for L = 10*n* + 5 (suitable for comparison with our models), we have only indirect evidence in support of their existence (see below).

### 3.2. Energy landscape

**Figure 4:**
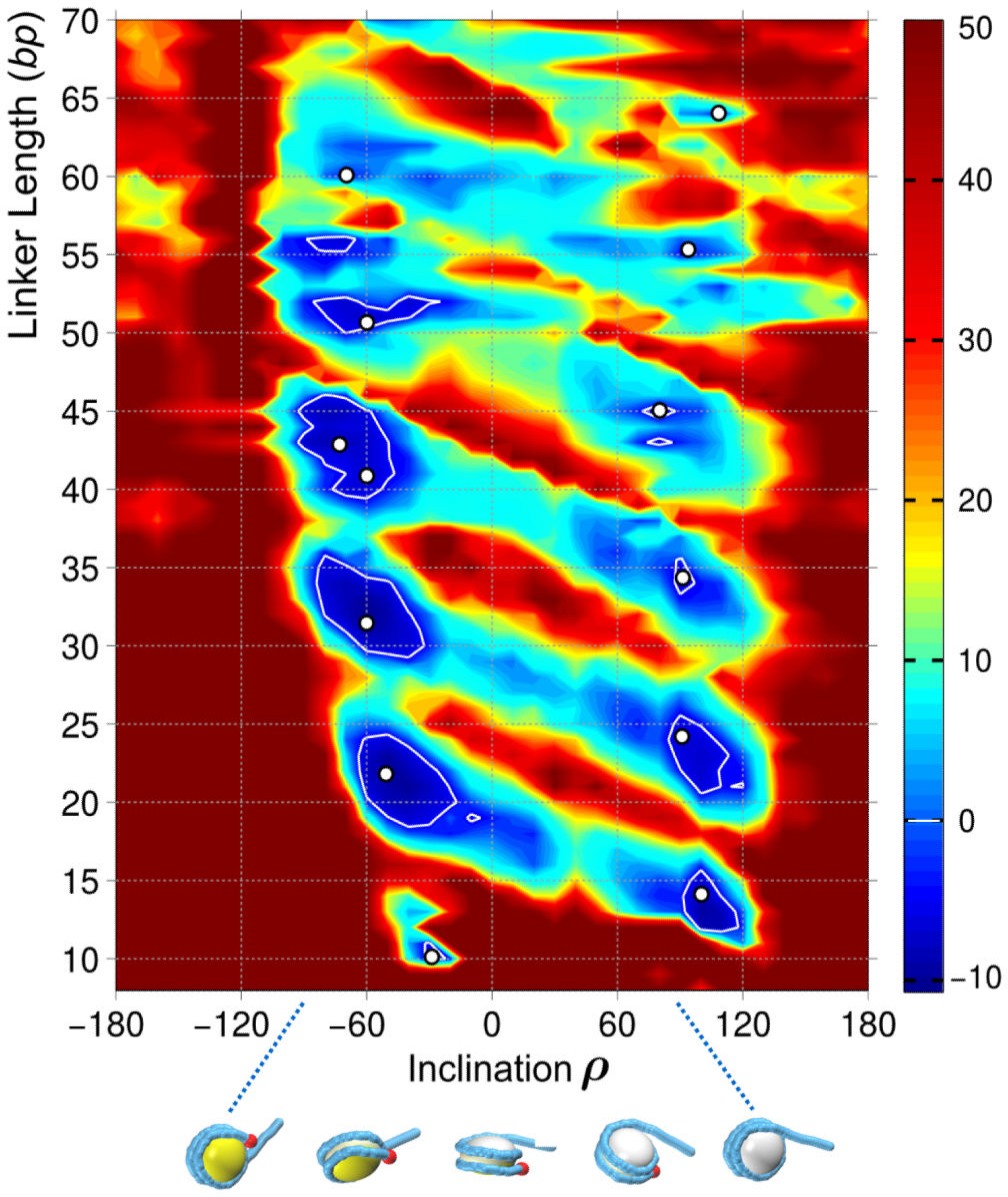
Total energy of the two-start fibers as a function of the inclination angle *ρ* and the linker length L Optimization is made in the space of three remaining fiber parameters, i.e., the radius, rise, and polar angle *ρ* (Figure 1). Dark blue regions represent stable structures, whereas dark brown areas are those with energies higher than 40 *kT*. Energies less than zero are highlighted by white contour lines. White circles show the positions of optimal structures separated by approximately a half pitch of B-DNA (5–6 bp). Note that for the relatively short linkers (up to 45 bp) there are two clearly visible energy minima (bimodality patterns described earlier [24]), which become “blurry” for the longer linkers. The two optimal regions of the inclination angle are marked by the white arrows and denoted T2 and T1 (see the bottom of the plot), corresponding to the two topological families of the fibers presented in Figure 3. *Bottom*: Different orientations of a nucleosome illustrating variation of the inclination angle *ρ* from −90° to +90°.

A more detailed presentation of our numeric results is given in a two-dimensional plot (*ρ*, L) with linker lengths varying continuously from ~ 10 to 70 bp, and the inclination angle *ρ* spanning the whole 360° interval (Figure 4). As in our earlier study with limited variation of the linker L [24], we observe two vertical rows of low energy regions (blue areas and white contour lines) arranged with 10–11 bp periodicity, which reflects the helical period of DNA duplex. The energy minima on the left side of the plot (−90° < *ρ* < −20°) correspond to the L = 10*n* values, whereas the minima on the right side (80° < *ρ* < 120° ) are observed for L = 10*n* + 5. In other words, for L = 10*n*, the “canonical” structures with negative angle *ρ* (similar to the fibers observed experimentally [7,8]; Figure 3A) are most favorable. They have the lowest energy values; thus, it is not surprising that they were stabilized and resolved by X-ray [7] and Cryo-EM [8] methods. By contrast, for L = 10*n* + 5, the “novel” configurations with positive angle *ρ* ≈ 90° are more preferable (see Figure 3B). Their energy is predicted to be less than that in the “canonical” structures, which is consistent with the observation that nucleosomal arrays with L = 10*n* + 5 are less prone to fold according to sedimentation measurements [16]. Accordingly, no experiment-based models of these structures are available to date.

The difference between the two types of fibers described above (“canonical” fibers for the {L = 10*n*} series and “novel” configurations for {L = 10*n* + 5}) is not limited to the inclination of nucleosomes. More importantly, the two families of structures are characterized by different DNA linking numbers (see the next section).

Finally, note that the increase in the linker length is accompanied by a gradual decrease in fiber stability (Figure 4). In particular, all energy values are positive for L > 57 bp, which generally agrees with the fact that two-start fibers are not formed when linkers are 50 bp or longer [9,10,41]. It is quite possible that the interdigitated one-start helix [9] is more favorable in this case (especially in the presence of linker histones).

### 3.3. DNA linking number in the chromatin fibers

In an early study by Simpson et al. [26] it was found that the number of nucleosomes reconstituted on ccDNA is approximately proportional to the number of superhelical turns formed in DNA. These results allowed considering the linking number ΔLk normalized per nucleosome, as a topological parameter characterizing spatial organization of the chromatin fiber independent of its length. However, a systematic analysis of the Δ*Lk* value as a function of the fiber length was never published (to the best of our knowledge). Below, we present the results of such an analysis for the two series of linker length, L = 10*n* and 10*n* + 5.

**Figure 5:**
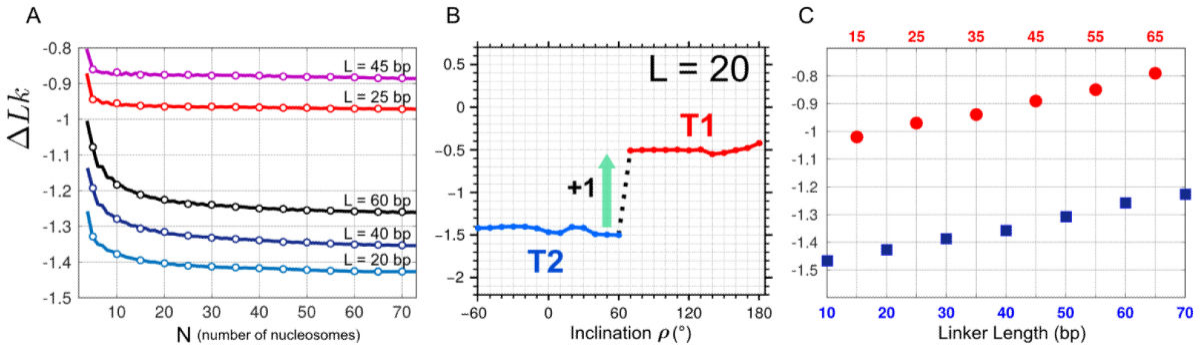
Linking number per nucleosome, Δ*Lk*, in regular fibers with various linker lengths (A) Dependence of Δ*Lk* on the number of nucleosomes for fibers with L = 10*n* = 20, 40, 60 bp, and L = 10*n* + 5 = 25, 45 bp. For every L, the energetically optimal fiber conformation was selected (Figure 4). (B) Schematic representation of the topological transition T2-T1 in the two-start fiber with L = 20 bp. Here Δ*Lk* is plotted *versus* changes in the inclination angle. Note that the linking number remains nearly constant except for a transition point where it abruptly changes by ~ 1. The transition point (here, *ρ* = 60°) depends on the linker length, L. For details see [24].(C) The limiting Δ*Lk* values (calculated for *N* = 100) are shown for the two series, L = {10*n*} (blue squares) and {10*n* + 5} (red circles). The color code is the same as in (B): the T1 type is shown in red and T2 in blue. Note that among the optimal fibers, the smallest Δ*Lk* = −1.7 is observed for L = 33 bp (not shown).

Figure 5A demonstrates the asymptotic behavior of Δ*Lk* depending on the number of nucleosomes in the fiber, *N*. We see that the linking number decreases significantly when *N* increases from 3 to 10, but then it converges rather fast, and Δ*Lk* remains practically the same for *N* > 30 (with the precision ~ 0.05). Note that this convergence is somewhat faster for the {10*n* + 5} series (L = 25 and 45 bp).

The curves presented in Figure 5A are naturally divided into two groups: for L = 10*n* + 5 the linking number is higher than −1, but for L = 10*n*, it is less than −1.2. This is consistent with our earlier observation of the topological polymorphism of two-start fibers [24]. We found that for all linker lengths there are two stereochemically feasible topoisomers (denoted T1 and T2) which have different linking numbers. Importantly, the optimal configurations of the fibers belong to different topological families, depending on the linker size: for L = 10*n* + 5 this is the topoisomer T1 that is most favorable, and for L = 10*n* this is the topoisomer T2. The two families of structures differ mostly by the inclination angle *ρ*.

The topological transition in the case of L = 20 bp is shown schematically in Figure 5B. This transition (associated with the change in inclination angle) is caused by an abrupt 360° change in the linker DNA twisting. The stereochemical details are presented elsewhere [24]; here we wish to emphasize that this T2-to-T1 transition (in the experimental system) requires the presence of nicking-closing enzyme such as topoisomerase I. (Naturally, this limitation is valid only for the DNA with closed ends.)

The two topological types, T1 and T2, are clearly separated in Figure 5C, where the limiting Δ*Lk* values (calculated for *N* = 100) are presented for the optimal configurations of the fibers with L = 10*n* and 10*n* + 5 bp. As in Figure 5A, the distinction between the T1 and T2 types is reflected in the linking number values, which are higher than −1.0 and lower than −1.2, respectively. An increase in the linker length is accompanied by a gradual decrease in the absolute value of Δ*Lk* such that for the “long” linkers L = 60–70 bp, Δ*Lk* is close to −1.2 (compared to −1.5 predicted for the “short” linkers L = 10–20 bp). At the same time, the difference ΔΔ*Lk* between the {10*n*} and {10*n* + 5} series remains approximately the same for the “long” and “short” linkers – that is, the difference between L = 20 and 25 bp, and between L = 60 and 65 bp, is expected to be ΔΔ*Lk* ≈ 0.5.

Before proceeding to the next section, we summarize the main results presented above:

(1) The finite size effect in calculating the DNA linking number proved to be significant if *N* < 10 (where *N* is number of nucleosomes in the fiber), but it can be practically neglected if *N* > 20. This is important for the quantitative interpretation of the topological gel assays used to estimate Δ*Lk* in SV40 minichromosome (*N* ≈ 20) [25,42,43] and in ccDNA containing 5S DNA repeats (*N* = 18) [26].

(2) Our classification of two-start chromatin fibers made for the relatively short linkers L < 40 bp [24] still holds for the linkers spanning the interval from ~ 10 to 70 bp. The energetically optimal fibers with L = 10*n* belong to the topological type T2, with Δ*Lk* varying from −1.2 to −1.4, while the fibers with L = 10*n* + 5 belong to the family T1, with Δ*Lk* varying from −1.0 to −0.8.

### 3.4. Relationship between nucleosome repeat length and gene expression level

**Figure 6:**
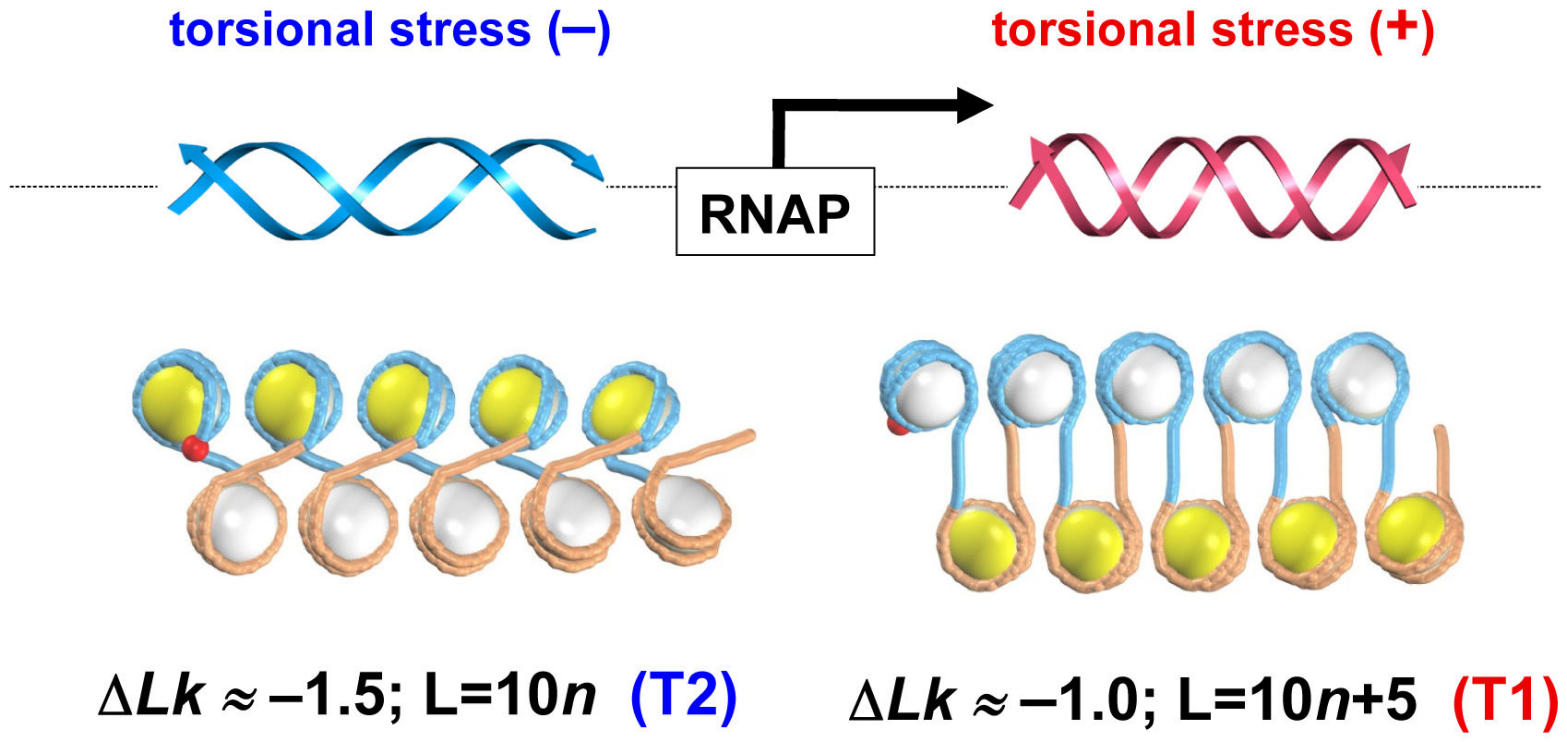
Chromatin fiber topology may be related to the level of gene expression in yeast A graphical illustration of the mechanism of transcription based on the twin domain model by Liu and Wang [44]. *Top*: The RNA polymerase (RNAP) is moving along DNA to the right, increasing the local DNA twisting ahead of the transcription complex and leaving undertwisted DNA behind. *Bottom*: The DNA torsional stress modulates the level of supercoiling, so that in the DNA regions upstream of RNAP, the DNA linking number becomes less (on average) than downstream from RNAP. The two fiber topoisomers, T1 and T2, are positioned in accord with this Δ*Lk* distribution. This model predicts that the T2 type fibers with the linker length L = 10*n* are stabilized upstream of RNAP, while the T1 fibers with L = 10*n*+5 are formed downstream from RNAP.

Potentially, the topological polymorphism of chromatin fibers described above can be utilized *in vivo* for regulation of transcription. Indeed, according to the twin domain model of Liu and Wang [44] the positive torsional stress propagates downstream and the negative stress accumulates upstream of the transcription complex (Figure 6A). The DNA torsional stress modulates the level of negative supercoiling of DNA *in vivo*: the positive stress decreases absolute level of DNA supercoiling, and negative stress increases it. Initially, this model was suggested for bacteria but later it was confirmed for eukaryotes as well [21,45].

Our observation of the two types of fibers (T1 and T2) having different linking numbers may be related to the transient DNA topological changes occurring during transcription. We suggest that the T1 topoisomer with Δ*Lk* ≈ −1 is formed predominantly downstream from RNA polymerase (Figure 6A), as opposed to the T2 topoisomer with Δ*Lk* ≈ −1.5, which is stabilized in the upstream regions (and more generally, in the regions with a low level of transcription). In this regard, it is important that the T1 and T2 topoisomers are characterized by distinct linker lengths (see above). Therefore, we expect that there might be a difference in the distribution of sizes of inter-nucleosome linkers in highly and lowly expressed genes.

To test this hypothesis, we analyzed the nucleosome positions [46] in ~ 3,500 baker’s yeast genes that are at least 1,000 bp long [47]. We selected 25% of genes that are highly expressed (with transcription rates from 4 to 200 [48]) and the 25% of genes that are lowly expressed (transcription rates 0.1–0.9). For brevity, we refer to these genes as UP- and DOWN-genes, respectively.

Distribution of nucleosome occurrences for UP- and DOWN-regulated genes reflects the difference in nucleosome repeat length (NRL) between these two sets of genes (Figure 6B). The peaks in the nucleosome distribution for UP-genes lag behind those for DOWN-genes, especially those that are far from the TSS—*e.g.,* peaks at 841 bp (UP) and 884 bp (DOWN). Moreover, the peaks in the distribution of the nucleosome occupancies for DOWN-genes are higher than those for UP-genes. This result is expected, because the higher number of nucleosomes slows transcription.

However, the following result is less trivial and thus more interesting: NRL ≈ 161 bp for the highly expressed genes and NRL ≈ 167 bp for the lowly expressed genes. In terms of the linker length, this means that L ≈ 14 bp for the UP-genes and L ≈ 20 bp for the DOWN-genes. Note that the genome-wide average for yeast is NRL = 162 bp, or L = 15 bp [12,14,15]; thus, the UP-regulated genes are more “typical” for yeast. This assessment agrees with the RNA-seq data [48] indicating that overall, the level of transcription in yeast is much more active than in higher eukaryotes. By contrast, DOWN-regulated genes can be considered as an exception in the case of yeast.

In summary, our results are consistent with the above hypothesis that nucleosomal arrays with L ≈ 10*n* + 5, which are characterized by a less negative superhelical density, are transcriptionally competent. Note also that the fibers with L ≈ 10*n* + 5 reveal a greater plasticity [16,24], which may facilitate formation of gene loops [49,50], thereby inducing transcription of the corresponding genes. Accordingly, in the silent (repressed) genes the predominant linker length is L ≈ 10*n*, which corresponds to a higher superhelical density and a higher stability of the chromatin fiber.

Naturally, this correlation between gene expression level and NRL raises many questions about the cellular mechanisms responsible for transient stabilization of one of the two fiber topoisomers. In particular, it would be interesting to see if there are any DNA sequence patterns distinguishing the two groups of genes (and the two types of nucleosome packaging). If the tendency observed for baker’s yeast also holds for other species, these intriguing questions should be the subject of a separate investigation.

## 4. Conclusion

We have expanded our earlier analysis of two-start chromatin fibers [24] to those with long linkers, up to L = 70 bp (NRL = 217 bp). The new results corroborate the main conclusion made earlier that fiber topology depends on the linker length L. For the series {L = 10*n*}, the energetically optimal topoisomers belong to the family T2, with the DNA linking number about −1.5, whereas for {L = 10*n* + 5}, the T1 topoisomers are more favorable, with Δ*Lk* ≈ −1.0 (Figure 3).

The calculated T2 forms closely match experimentally observed structures for L = 20, 30 and 40 bp. The energy map (Figure 4) suggests that the two-start fibers for “long” linkers L = 50, 60 and 70 bp are relatively unstable (compared to L = 20–40 bp). This is consistent with Routh et al. [9] data indicating that for L = 50 bp, the chromatin fibers are not formed without linker histone. (When the histone H5 is added, an alternative structure is formed, probably an interdigitized one-start superhelix, computational analysis of which goes beyond the limits of this study.)

By contrast, the predicted T1 topoisomers are drastically different from the known fiber structures [7,8]. Locally, the T1 and T2 forms are characterized by different inclination of the nucleosomal disks (Figure 3). In terms of “global” organization of DNA, these forms comprise two distinct topological families with different DNA linking numbers (Figure 5). Conformational transition between the T1 and T2 topoisomers is possible only in the presence of nicking-closing enzymes (provided that the DNA ends are restrained). Note that our computations agree with the finding of Correll et al. [16] that the fibers with L = 10*n* + 5 = 25 bp are less stable than the fibers with L = 10*n* = 20 and 30 bp. This may be one of the reasons why the “new” topoisomer T1 (with L = 10*n* + 5) was not observed earlier.

In addition, we tested our prediction [24] concerning the topological difference between the two types of fibers with L = 20 and 25 bp. Our approach is similar to that described by Simpson et al. [26]; the main difference is that in our case these are tandem arrays of the “601” sequences [11] instead of the 5S DNA repeats used earlier [26]. Our preliminary results support the predicted difference of ΔΔ*Lk* ≈ 0.5 between the two topoisomers (T. Nikitina, D.N., S.A. Grigoryev and V.B.Z., unpublished observation). We consider this as a proof of principle and anticipate that using topological gel assays will allow us to make a detailed quantitative comparison between the two types of chromatin fiber organization.

For the first time, we have systematically analyzed how the finite size of chromatin fibers affects the calculated DNA linking number. Our results are important for numerical interpretation of the topological gel assays made for strongly positioned nucleosomes such as “601” [11] or 5S DNA [26]. In particular, if the array contains N ≈ 5 positioned nucleosomes, the DNA linking number differs from its “ideal” limiting value by 0.1 or even by 0.2, depending on the linker length (Figure 5A). Therefore, appropriate adjustments are necessary to account for the size of the nucleosomal array.

The same reasoning is applicable to the nucleosome positioning *in vivo*. In yeast, a typical nucleosomal organization is represented by relatively short clusters of N ≤ 5 nucleosomes, separated by gaps [15,51,52]. Therefore, instead of using the “ideal” Δ*Lk* values which are correct only for “long” arrays, one has to consider the finite size effect described in Figure 5A.

Earlier, we hypothesized [24] that the different topology and flexibility of the two types of fibers (T2 with L = 10*n* and T1 with L = 10*n* + 5 bp) may be utilized by cells in functionally distinct parts of the genome. In particular, we suggested that highly and lowly expressed genes may have different L values (on average). Here, we present data for yeast supporting this hypothesis (Figure 6B). It would be interesting to see whether this topological mechanism of transcription regulation is applicable to higher eukaryotes as well. For example, the data on genome-wide nucleosome positioning provided by Teif et al. [53] for three murine cell lines suggests that the average NRL (and the linker length L) are changed upon cell differentiation. Note, however, that the higher eukaryotic chromatin contains significant amounts of linker histone H1, and the NRL strongly depends on this amount [54,55]. Therefore, analysis of a possible interplay between DNA topology and the level of transcription has to take into account the role played by histone H1 in electrostatic neutralization of the linker DNA and stabilization of chromatin fiber [56].

We consider the topological mechanism accounting for distinct organization of transcriptionally active chromatin not instead of, but rather in addition to the alternative mechanisms proposed earlier, such as partial unwrapping of nucleosomal DNA due to the loss of histones H2A-H2B [57] or formation of “reverse nucleosomes” [58,59] in the course of transcription (see recent review by Teves and Henikoff [60]).

Our results may reflect a more general tendency of chromosomal domains containing active or repressed genes to retain topologically distinct higher-order structures. Indeed, transcriptionally silent domains were shown to acquire a stable negative supercoiling [61], which agrees with our findings: (i) the computational prediction [24] that {10*n*} chromatin is more negatively supercoiled than {10*n* + 5} chromatin; and (ii) the predominant occurrence of linker L ≈ 10*n* in genes with a low level of expression (Figure 6B).

In this regard, it is remarkable that histone acetylation reduces the absolute value of DNA linking number from 1.0 to 0.8 [27]. The histone acetylation is one of the epigenetic markers indicating the increased level of transcription. Therefore, the observation made by Norton et al. [27] fits nicely into our hypothesis that the reduced level of DNA supercoiling correlates with the high level of transcription.

## Supplementary Material

1

## Figures and Tables

**Figure 7:**
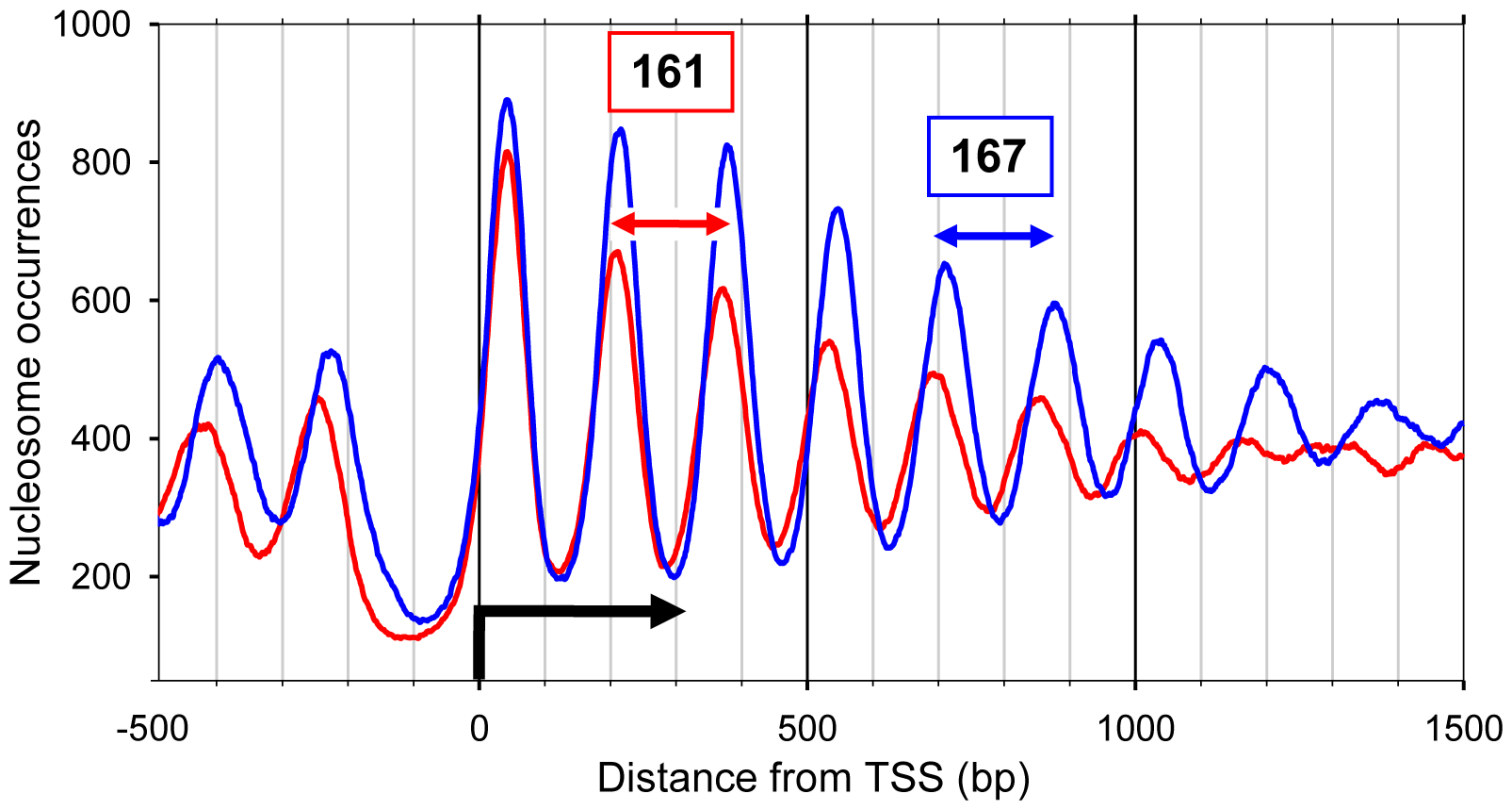
Nucleosome occurrences for two groups of yeast *S. cerevisiae* genes Positions of nucleosome dyads relative to the transcription start site (TSS) are calculated for nucleosome fragments 147–152 bp in length [15,46]. Note the nucleosome-depleted region upstream from TSS [64]. The red curve represents the 25% of genes that are highly expressed (UP-genes) and the blue curve shows the 25% of genes that are lowly expressed (DOWN-genes). The nucleosome occurrences are normalized by the number of genes in each set (i.e., 860 genes). The running averages of 51 bp are shown. Averaging the distances between the peaks, we obtained NRL = 161.2 (±3.5) for UP-genes and NRL = 167.2 (±2.9) for DOWN-genes.
