# Supplementary material for: Topological diversity of chromatin fibers: Interplay between nucleosome repeat length, DNA linking number and the level of transcription
Source: AIMS Biophys. Author manuscript; Available in PMC 2017 Jan 27. (PMC5271602; doi:10.3934/biophy.2015.4.613)
Supplement: 1 [file NIHMS809802-supplement-01.pdf]

AIMS Biophysics

http://www.aimspress.com/

AIMS Biophysics, 2(4): 613-629.
DOI: 10.3934/biophy.2015.4.613
Received date 27 September 2015,
Accepted date 28 October 2015,
Published date 03 November 2015

Research article

Topological diversity of chromatin fibers: Interplay between nucleosome repeat length, DNA linking number and the level of transcription

Davood Norouzi [1], Ataur Katebi [1], Feng Cui [2], and Victor B. Zhurkin [1,*]

[1] Laboratory of Cell Biology, National Cancer Institute, NIH Bethesda, MD 20892, USA
[2] Thomas H. Gosnell School of Life Sciences, Rochester Institute of Technology, 85 Lomb Memorial Drive, Rochester, NY 14623, USA

* **Correspondence:** Email: zhurkin@nih.gov.

Supplementary

For L = 20 bp, our structure has the inclination angle $\rho = -45°$ and rise = 27 Å. The "direct" model by Schalch et al. [7] is based on the X-ray tetranucleosome structure; it has inclination about −50°/−40° and low rise = 17 Å. Note, however, that this model has "steric overlaps" [7]; the authors suggest that these overlaps can be relieved by increasing the separation of tetranucleosomes. On the other hand, using the EM images for L = 20 bp [9,16], one can estimate the rise to be ~ 25 Å, which is close to 27 Å in our energetically optimal fiber (**A**).

For L = 30 bp, Song et al. [8] built the high-resolution model based on the Cryo-EM data, with the inclination angle $\rho \approx -60°$ and rise = 24 Å (**E**). Our optimal structure is very close to this model: $\rho = -60°$ and rise = 25 Å (**B**).

For L = 40 bp, the Cryo-EM-based model has $\rho \approx -60°$ and rise = 22.5 Å, compared to $\rho = -70°$ and rise = 20 Å in our optimal structure (**C**). Note that in both the theoretical (**B, C**) and the Cryo-EM-based models (**E, F**) the linker increase from L = 30 to 40 bp is accompanied by a decrease in superhelical rise and increase in the diameter of fibers.

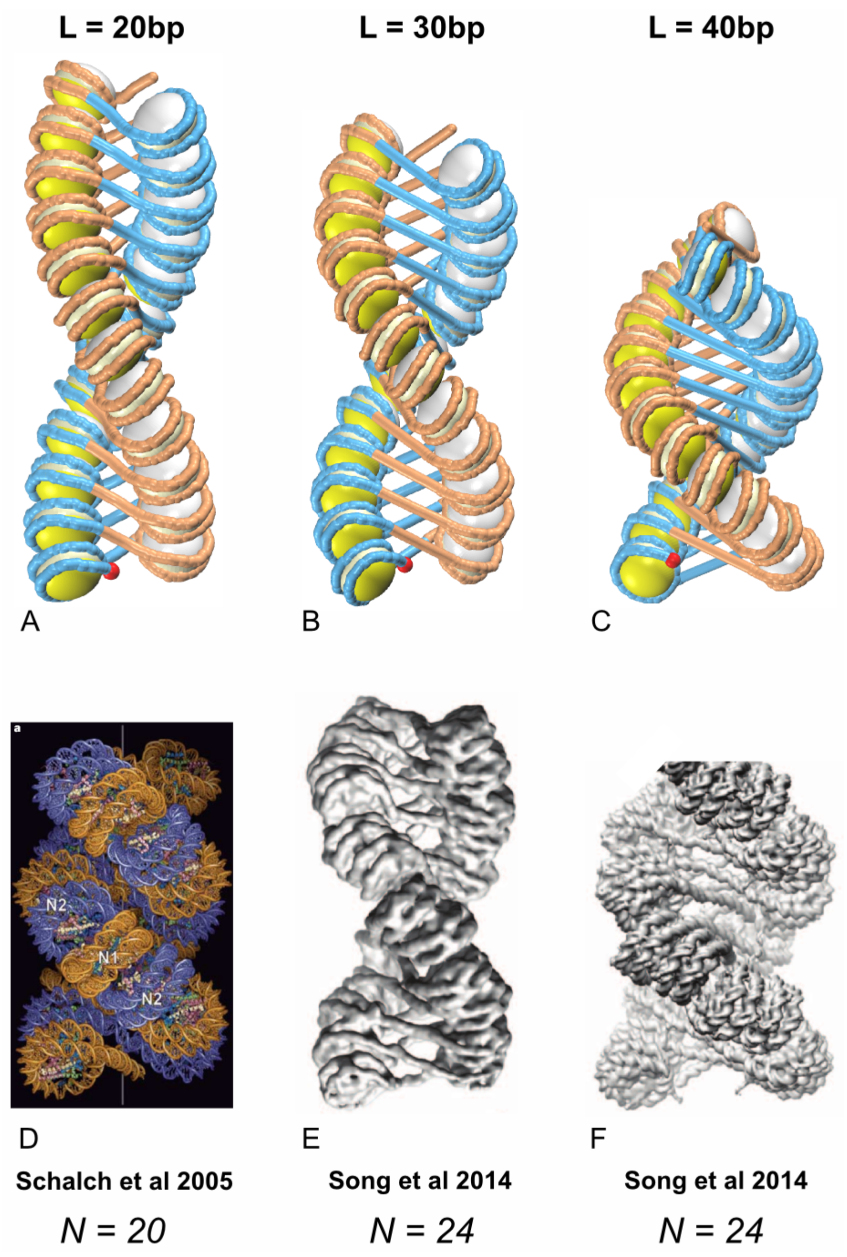

Figure S1. Comparison of the energetically optimal fibers with the experiment-based models. The computed structures for L=20, 30, 40 bp (A, B, C) are the same as shown in Figure 3. In each case, the number of nucleosomes, N, is 24. (D–F) The published experiment-based models are shown for comparison, with the N values given below.

AIMS Press

© 2015 Victor B. Zhurkin, et al., licensee AIMS Press. This is an open access article distributed under the terms of the Creative Commons Attribution License (http://creativecommons.org/licenses/by/4.0)
